# 48 Genital Burns Are Associated with Worse Psychosocial and Physical Outcomes

**DOI:** 10.1093/jbcr/irae036.072

**Published:** 2024-04-17

**Authors:** Suhaib Shah, Carolina Segura, Yash Ramgopal, Christopher G Richter, Sunskruthi Krishna, Maria Haseem, Mathilda Nicot-Cartsonis, Georgiy Golovko, Steven E Wolf, Juquan Song, Amina E I ayadi

**Affiliations:** University of Texas Medical Branch, Galveston, TX; University of Texas Medical Branch, Galveston, Texas; University of Texas Medical Branch at Galveston, Galveston, TX; University of Texas Medical Branch, Blocker Burn Unit, Galveston, TX; University of Texas Medical Branch, Galveston, TX; University of Texas Medical Branch, Galveston, Texas; University of Texas Medical Branch at Galveston, Galveston, TX; University of Texas Medical Branch, Blocker Burn Unit, Galveston, TX; University of Texas Medical Branch, Galveston, TX; University of Texas Medical Branch, Galveston, Texas; University of Texas Medical Branch at Galveston, Galveston, TX; University of Texas Medical Branch, Blocker Burn Unit, Galveston, TX; University of Texas Medical Branch, Galveston, TX; University of Texas Medical Branch, Galveston, Texas; University of Texas Medical Branch at Galveston, Galveston, TX; University of Texas Medical Branch, Blocker Burn Unit, Galveston, TX; University of Texas Medical Branch, Galveston, TX; University of Texas Medical Branch, Galveston, Texas; University of Texas Medical Branch at Galveston, Galveston, TX; University of Texas Medical Branch, Blocker Burn Unit, Galveston, TX; University of Texas Medical Branch, Galveston, TX; University of Texas Medical Branch, Galveston, Texas; University of Texas Medical Branch at Galveston, Galveston, TX; University of Texas Medical Branch, Blocker Burn Unit, Galveston, TX; University of Texas Medical Branch, Galveston, TX; University of Texas Medical Branch, Galveston, Texas; University of Texas Medical Branch at Galveston, Galveston, TX; University of Texas Medical Branch, Blocker Burn Unit, Galveston, TX; University of Texas Medical Branch, Galveston, TX; University of Texas Medical Branch, Galveston, Texas; University of Texas Medical Branch at Galveston, Galveston, TX; University of Texas Medical Branch, Blocker Burn Unit, Galveston, TX; University of Texas Medical Branch, Galveston, TX; University of Texas Medical Branch, Galveston, Texas; University of Texas Medical Branch at Galveston, Galveston, TX; University of Texas Medical Branch, Blocker Burn Unit, Galveston, TX; University of Texas Medical Branch, Galveston, TX; University of Texas Medical Branch, Galveston, Texas; University of Texas Medical Branch at Galveston, Galveston, TX; University of Texas Medical Branch, Blocker Burn Unit, Galveston, TX; University of Texas Medical Branch, Galveston, TX; University of Texas Medical Branch, Galveston, Texas; University of Texas Medical Branch at Galveston, Galveston, TX; University of Texas Medical Branch, Blocker Burn Unit, Galveston, TX

## Abstract

**Introduction:**

Genital burns (GB) represent a specialized subset of injuries with a profound impact on quality of life and psychosexual health. Analysis of GB is notably sparse in the current literature. This study aims to address GB short and long-term outcomes, psychosocial outcomes, and outcome variations based on the gender. We compared GB with burns in general, controlling for the extent and severity of injury.

**Methods:**

Using a federated network of real-world data, GB (n = 3,508) and all burn (AB) (n = 358,136) cohorts were created using specific ICD-10 codes. Cohorts were balanced using age at index, gender, race, and ethnicity. Covariates were defined by a time window up to 1 day before burn. The following outcomes were selected for short-term analysis (1 month): deceased, PTSD, depression, anxiety, ICU admit, skin graft loss, sepsis, pneumonia, respiratory failure, acute renal failure, and ventilator use. Long-term analysis (5 years) was performed for the following outcomes: deceased, PTSD, depression, anxiety, joint pain, chronic neuropathic pain, and pruritus. For the gender comparisons, catheter infection, UTI, and dysuria were also investigated. We ran measures of association cohort analyses to compare outcomes with risk ratios (RR) and 95% confidence intervals (95% CI).

GB (balanced: n = 3,277) was compared to AB (balanced: n = 3,277). To focus on isolated area burns, we compared non-severe (TBSA < 20%) GB (n = 1,918) to non-severe AB (n = 101,446) (balanced cohorts: n = 1,896 each). Lastly, non-severe male GB (n = 2,353) was compared to non-severe female GB (n = 1,183) (balanced cohorts n = 649 each).

**Results:**

When comparing all GB and all AB patients a substantial array of differences emerged: In non-severe burns, GB patients experienced significantly higher RRs for death (RR = 2.36, 95% CI: 1.171-4.77), anxiety (RR = 2.3, 95% CI: 1.2-4.41), hospitalization (RR =1.58, 95% CI: 1.301-1.919), ventilator use (RR = 2.087, 95% CI: 1.275-3.417), and pruritus (RR = 1.969, 95% CI:1.08-3.587) in the short-term, and higher rates of pruritus (RR = 1.881, 95% CI: 1.36-2.603) and neuropathic pain (RR = 1.827, 95% CI: 1.157-2.885) in the long-term.

Gender-based GB comparisons showed significant short-term differences including an increased rate of anxiety (RR = 0.314, 95% CI: 0.154-0.639) in females. Long-term outcomes showed a higher rate of anxiety (RR = 0.722, 95% CI: 0.523-0.998), dysuria (RR = 0.561, 95% CI: 0.378-0.831), and UTI (RR = 0.394, 95% CI: 0.277-0.56) in female patients.

**Conclusions:**

Genital burn patients have higher risks of developing certain short- and long-term outcomes, some of these being more pronounced based on gender.

**Applicability of Research to Practice:**

Increased rates of death, anxiety, hospitalization, pruritus, and neuropathic pain amongst genital burn patients underlies the need for nuanced clinical and psychosocial approaches, specifically tailored to address the unique challenges and requirements associated with genital burns.

